# Transcriptomics analysis revealed that TAZ regulates the proliferation of KIRC cells through mitophagy

**DOI:** 10.1186/s12885-024-11903-9

**Published:** 2024-02-19

**Authors:** Zhen He, Jianxi Shi, Bing Zhu, Zhentao Tian, Zhihong Zhang

**Affiliations:** 1 Department of Urology, Tianjin Institute of Urology, The Second Hospital of Tianjin Medical University, Tianjin, China; 2 Department of Urology, First Teaching Hospital of Tianjin University of Traditional Chinese Medicine, National Clinical Research Center for Chinese Medicine Acupuncture and Moxibustion, Tianjin, China

**Keywords:** Renal clear cell carcinoma (KIRC), Transcriptional co-activator with PDZ-Binding motif (TAZ), The Cancer Genome Atlas (TCGA), Mitophagy

## Abstract

Transcriptional Co-Activator with PDZ-Binding Motif (TAZ, also known as WWTR1) is a downstream effector of the Hippo pathway, involved in the regulation of organ regeneration and cell differentiation in processes such as development and regeneration. TAZ has been shown to play a tumor-promoting role in various cancers. Currently, many studies focus on the role of TAZ in the process of mitophagy. However, the molecular mechanism and biological function of TAZ in renal clear cell carcinoma (KIRC) are still unclear. Therefore, we systematically analyzed the mRNA expression profile and clinical data of KIRC in The Cancer Genome Atlas (TCGA) dataset. We found that TAZ expression was significantly upregulated in KIRC compared with normal kidney tissue and was closely associated with poor prognosis of patients. Combined with the joint analysis of 36 mitophagy genes, it was found that TAZ was significantly negatively correlated with the positive regulators of mitophagy. Finally, our results confirmed that high expression of TAZ in KIRC inhibits mitophagy and promotes KIRC cell proliferation. In conclusion, our findings reveal the important role of TAZ in KIRC and have the potential to be a new target for KIRC therapy.

**Supplementary Information:**

The online version contains supplementary material available at 10.1186/s12885-024-11903-9.

## Introduction

Renal cell carcinoma (KIRC) is the sixth most common cancer in men and the tenth most common cancer in women worldwide [1]. So far, the subtypes of KIRC are mainly divided into clear cell carcinoma, papillary carcinoma and chromophobe carcinoma. The incidence of KIRC is increasing year by year. Most KIRCs are detected incidentally, but a significant number develop locally advanced disease or even distant metastases [2]. KIRC is the 13th most common cause of cancer death worldwide, according to the latest figures from the World Health Organization [3]. KIRC has become a major challenge to human health, and it is urgent to explore the mechanism of its occurrence and development.

Mitophagy, as a type of selective autophagy, is selective autophagy to remove defective mitochondria, and is one of the most important mechanisms for cells to maintain healthy mitochondria. Autophagy is often involved in the regulation of cell death and, among other things, can affect or regulate processes such as inflammation, innate immunity, and host defense [4, 5]. Studies have shown that autophagy is associated with various kidney diseases such as acute kidney injury, chronic kidney disease and kidney cancer. It has been shown that low expression of melanoma-deficient protein 2 (AIM2) in KIRC is associated with poor prognosis, whereas high expression of AIM2 was demonstrated to enhance the expression of autophagy-associated genes Bcl-2, Beclin-1, LC3-II, and ATG-5 in KIRC cells, and it has been found that blockade of autophagy by 3-methyladenine (3-MA) abrogated the suppression of cell migration and invasion by overexpression of AIM2, suggesting that AIM2 inhibits malignant behavior of KIRC by enhancing autophagy [6]. At the same time, autophagy also has great research potential in the treatment of KIRC. Autophagy-modulating compounds such as everolimus (an mTOR inhibitor and autophagy activator) and hydroxychloroquine (an autophagy inhibitor) have recently been used in phase I/II trials in combination therapy for advanced KIRC [7]. In summary, autophagy is closely linked to the proliferation and invasive ability of KIRC cells and has a greater research prospect in the treatment of KIRC.

Transcriptional Co-Activator with PDZ-Binding Motif (TAZ, also known as WWTR1), similar to Yes-associated Protein (YAP), is a downstream effector of the Hippo pathway and is required for the regeneration of different organs [8]. Excessive activation of TAZ is now widely believed to promote cancer development. One study showed that YAP/TAZ promotes glycolysis and NF2-deficient KIRC growth through transcriptional and PI3K-AKT signaling pathways. The study also found that combined inhibition of YAP/TAZ and MEK durably blocked the growth of NF2-deficient KIRC cells [9]. Yang et al. showed that TAZ regulates ferroptosis in KIRC cells by modulating EMP1-NOX4-mediated cell density [10]. In addition, TAZ is also closely related to mitophagy. Fan et al. found that PINK1-dependent mitophagy regulated the migration and homing of multiple myeloma cells by activating the MOBIB-mediated Hippo-YAP/TAZ pathway [9]. Therefore, the role of TAZ in KIRC deserves our further exploration.

In recent years, the incidence of KIRC is increasing year by year, and the study of the biological mechanisms and therapeutic strategies of KIRC is an urgent scientific problem. Recent studies have shown that autophagy is closely related to KIRC, and there is evidence that autophagy-related genes and regulators have a significant impact on the biological behavior of KIRC. Mitophagy, as a type of autophagy, is a promising research direction in the field of KIRC. TAZ is a mitophagy-related gene and is closely related to KIRC. Therefore, our study mainly explores the potential mechanism of TAZ and mitophagy in KIRC.

## Materials and methods

This experiment does not involve any human experimentation and the use of human tissue samples. All procedures conducted in this study using human data were in accordance with the Declaration of Helsinki. All experimental protocols in this study were approved by the Research Ethics Committee of The Second Hospital of Tianjin Medical University.

### Collection and progression of public data

The 36 genes related to mitophagy were obtained from the molecular signature database (MSigDB). Obtained KIRC’s mRNA expression profile data (https://xena.ucsc.edu/) and clinical data from the TCGA database. The expression profile and clinical data of E-MTAB-1980 renal carcinoma was obtained from the Array Express database. TCGA-KIRC includes 535 tumor samples and 72 normal tissue samples, E-MTAB-1980 includes 100 tumor data. TCGA-KIRC mRNA expression values were normalized and converted to log2(TPM + 1) format. E-MTAB-1980 data for subsequent model validation.

### Screening of differentially expressed genes and prognostic genes

The limma package (https://www.bioconductor.org/) was used to compare the tumor and normal data in TCGA-KIRC and calculate the differential expression analysis. Genes with|LogFC|>1 and corrected P-value < 0.05 were defined as differentially expressed genes. The KIRC differentially expressed genes and mitophagy-related genes were intersected, and finally 9 overlapping mitophagy-related differential genes were screened out. To further explore the clinical guiding value of differential genes, we matched mitophagy-related genes in TCGA-KIRC with clinical data, and performed univariate cox regression analysis using the survival package, and the log-rank test evaluated the difference in prognosis, and mitophagy-related genes with a P value less than 0.05 were defined as KIRC prognosis-related genes. Further use the GEPIA (http://gepia.cancer-pku.cn/) website to draw Kaplan-Meier survival curves of prognosis-related genes to verify the ability of genes to affect patient prognosis.

### Interaction of mitophagy-related genes

Submit nine KIRC prognostic-related mitophagy genes to STRING website (https://www.string-db.org/) for protein interaction analysis, and draw a protein-protein interaction network diagram. Extract the mRNA expression data of these 9 genes, and use the rcorr function in the Hmisc package to calculate the correlation of the 9 genes at the mRNA level based on the Pearson method, and explore co-expressed genes. Download 50 cancer-related Hallmark pathways in the (MSigDB), use the GSVA package to calculate the activity of 50 pathways in KIRC, further match with the mRNA expression data of 9 genes, and calculate the correlation between the 9 mitophagy-related genes (MPRGs) and 50 pathway activities, so as to explore the underlying molecular mechanism of MPRGs.

### Establishment and validation of a prognostic model for MPRGs

Combine the expression data of prognostic MPRGs with clinical data, use Lasso-penalized Cox (LASSO-Cox) regression analysis to exclude genes with overfitting tendency, and construct a prognostic model with the glmnet R package. A risk model was constructed in TCGA-KIRC using LASSO regression analysis, and a risk score was obtained using the obtained coefficients and gene expression values. The risk score formula is as follows: ${\rm{Riskscore = sum (Ex}}{{\rm{p}}_{{\rm{gene}}}}{\rm{*coef)}}$.

The ideal cut off value of genes were determined by survey point R function based on survey R package, then the samples were divided into high -/low expression group to depot Kaplan Meier survival curve. Use the surv_cutpoint function in the survminer package to calculate the optimal cut-off value for the risk score, and divide the TCGA-KIRC data into high-risk and low-risk groups. The predictive power of the prognostic models was assessed using the plotting of Kaplan-Meier survival curves and Receiver operating characteristic (ROC) curve analyzes (“survivalROC” package). ROC curves were quantified using the area under the curve (AUC). The expression of related genes in the high and low risk groups is displayed with a heat map. The same analysis was performed in the E-MTAB-1980 data to test the generalizability of this risk score. In order to further study the robustness of the risk prediction model, we applied the risk score to different subgroups of clinical factors, and drew Kaplan-Meier survival curves for validation.

### Build a nomogram model

Univariate and multivariate Cox regression analyzes were performed on the risk score and clinical variables in TCGA-LIHC and E-MTAB-1980, and the results showed that the risk score was an independent risk factor in the univariate and multivariate Cox regression (*P* < 0.05). We used the “rms” R package to build a nomogram model for predicting prognosis, which provided more accurate prognosis predictions for clinical patients based on risk scores and clinical characteristics. In addition, the area under the ROC curve (AUC) and the calibration plot were used to estimate the discrimination accuracy. AUC value greater than 0.7 is a reasonable estimate. Decision curve analysis (DCA) was then used to evaluate the clinical utility of the nomogram model.

### Cell culture and reagents

Human renal cortical proximal tubule epithelial cell (HK2) and human renal cancer cells (A498 and 786-O) were obtained from the Shanghai Cell Bank of the Type Culture Collection Center of the Chinese Academy of Sciences. HK2 was cultured in DMEM (HyClone, USA) supplemented with 10% fetal bovine serum (HyClone, USA) and penicillin streptomycin (100 units/ml) in a 37 ° C and 5% CO2 incubator. A498 in an incubator at 37 °C and 5% CO2 in MEM (HyClone, USA) supplemented with 10% fetal bovine serum (HyClone, USA) and penicillin-streptomycin (100 units/ml) cultivated in. 786-O in an incubator at 37 °C and 5% CO2 in RPMI-1640 (HyClone, USA) supplemented with 10% fetal bovine serum (HyClone, USA) and penicillin-streptomycin (100 units/ml) cultivated in.

### siRNA molecule transfection

siRNA was purchased from Suzhou Gemma Gene Co., Ltd. Cultivate the experimental cells in the rapid growth phase to an appropriate number and density, and transfect siRNA and non-target siRNA (disruption control) into the cells according to the experimental procedure of the manufacturer’s instructions. Cells were cultured for 48 h after transfection, and then related experiments were performed. Sequences of TAZ-siRNA and Scramble siRNA were as follows (5′-3′): TAZ-siRNA#1 GCUCAUGAGUAUGCCCAAUTT; TAZ-siRNA#2 GGACAAACACCCATGAACA; TAZ-siRNA#3 GGUACUUCCUCAAUCACAUTT, and Scramble siRNA UUCUCCGAACGUGUCACGUTT (GenePharma Co., Ltd., Suzhou, China).

### JC-1 assay

The experiment uses the mitochondrial membrane potential detection kit (JC-1) (Beyotime, China), JC-1 is an ideal probe widely used to detect the mitochondrial membrane potential. When the mitochondrial membrane potential is high, it produces red fluorescence; when the mitochondrial membrane potential is low, it produces green fluorescence.

### ATP detection

The ATP content in the experimental samples was detected using the ATP detection kit (Beyotime, China) according to the experimental procedures in the manufacturer’s instructions. ATP content normalized to cell number.

### 10 ADP/ATP ratio

The content of adenosine diphosphate (ADP) and adenosine triphosphate (ATP) in the experimental samples was determined using the ADP/ATP Ratio assay kit (Abnova, Wu Han, China). (A) Release ADP and ATP in the cells: use the working solution in the kit to lyse the cells, ATP can react with the substrate D-luciferase to generate fluorescence, and measure the concentration of ATP in the cells according to the fluorescence intensity; (B) ADP is converted into ATP by enzymatic reaction, and then measured again by step A.

### 11 western blot analysis

First, extract the proteins of various renal cell carcinoma cells (HK2, A498, 786-O), and use the BCA method to determine the protein concentration. An equal amount of protein was added to a 10% polyacrylamide gel electrophoresis plate (SDS-PAGE), followed by electrophoresis, membrane transfer and blocking. Based on the kDa value of the target band and the Marker band as a reference, the region where the target protein is located is cut with a width of about 1 cm. Then, the cut band is subjected to subsequent operations such as applying primary and secondary antibodies. Finally, use ECL luminescent liquid to expose and take pictures. Antibodies TAZ and GAPDH used in this study were purchased from Proteintech Wuhan.

### 12 statistical analysis

Differences between the two groups were compared using unpaired t-test. Univariate and multivariate Cox regression analysis was used to analyze the relationship between each variable and the prognosis of patients. The log-rank test assessed the difference in prognosis. In all analyses, p-values < 0.05 were considered statistically significant. The data of this experiment are shown as mean ± SEM of three independent experiments, n.s., no significant; *, P-value < 0.05; **, P-value < 0.01; ***, P-value < 0.001; ****, P-value < 0.0001.

## Result

### Mitophagy-related genes (MRGs) differential expression and prognosis analysis

**Fig. 1:**
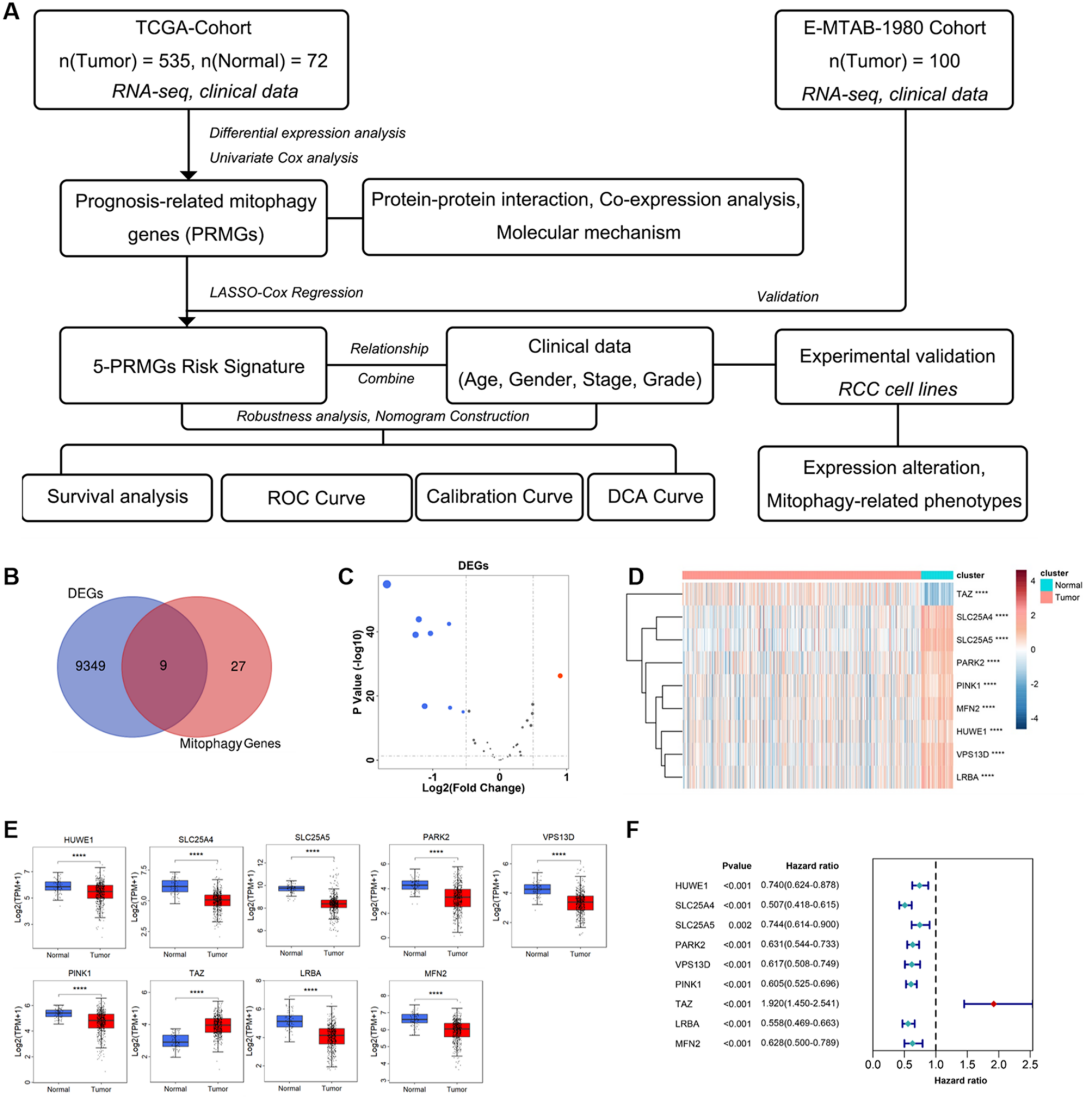
MRGs differential expression and prognosis analysis. (**A**) Schematic diagram of the research; (**B**) Venn diagram analysis revealed differentially expressed mitophagy genes in KIRC; (**C**) Volcano plot of changes in mitophagy gene expression, red indicates up-regulation, blue indicates down-regulation, and gray indicates no expression change; (**D**) Heat map of differentially expressed genes in KIRC and normal tissues; (**E**) Boxplot of differentially expressed genes in KIRC and normal tissues; (**F**) The forest plot showed the relationship between the differential genes related to mitophagy and the prognosis of KIRC patients. The red dots indicate that the gene is a risk factor for prognosis, and the green dots indicate that the gene is a protective factor for prognosis

The flow chart of this study is shown in Fig. 1A. We first use the limma R-package to calculate the differentially expressed genes (DEGs) of TCGA-KIRC, and take the intersection with mitophagy-related genes (the screening condition is that the absolute value of LogFC is greater than 0.5, and the corrected P value is less than 0.05). We found that 9 mitophagy-related genes were differentially expressed in KIRC (Fig. 1B and C). Among them, TAZ was up-regulated in tumor tissue compared with para-cancerous tissue, and the expression of the remaining eight genes (including SLC25A4, SLC25A5, PARK, PINK1, MFN2, HUWE1, VPS13D, and LRBA) was down-regulated in tumor tissue (Fig. 1D and E). Further univariate cox regression analysis, we found that all MRGs were associated with the overall survival of KIRC patients, these genes we called mitophagy prognosis-related genes (MPRGs). Interestingly, TAZ was a risk factor for the prognosis of KIRC patients, and the remaining 8 down-regulated genes were protective factors for the prognosis of KIRC patients (Fig. 1F). Survival analysis of KIRC patients was performed using the GEPIA website. Based on the median value of gene expression, patients were divided into high and low expression groups and Kaplain-Meier survival curves were drawn. Consistent with Fig. 1F, patients with high expression of TAZ had a poorer prognosis, while patients with high expression of the remaining eight genes had a better prognosis (Figure S1).

### Cross-talk and molecular mechanism between MPRGs

**Fig. 2:**
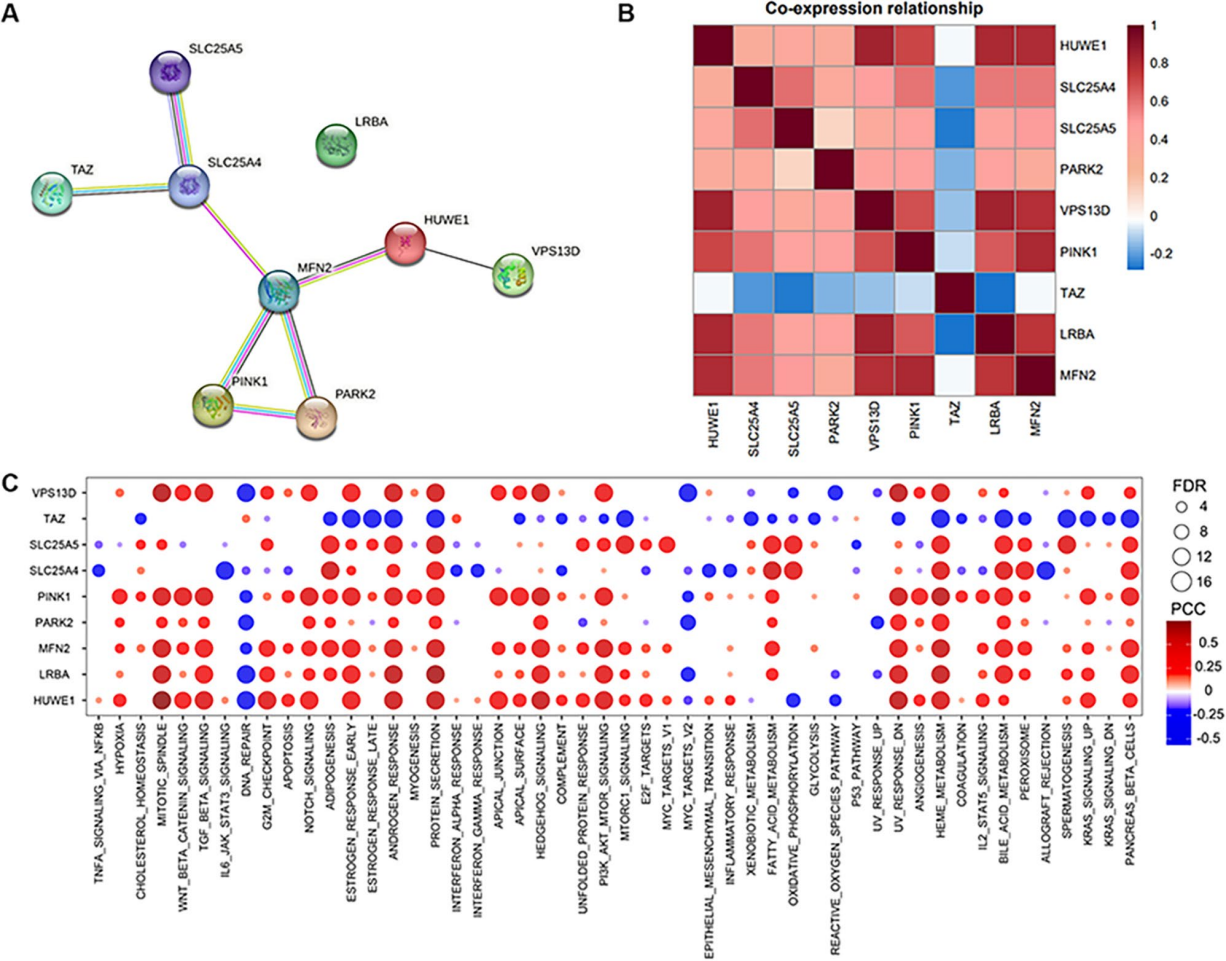
Cross-talk and molecular mechanism between MPRGs. (**A**) Protein-protein interaction (PPI) network diagram of mitophagy genes; (**B**) The heat map showed the co-expression relationship between mitophagy-related genes on mRNA, red is positive correlation, blue is negative correlation; (**C**) The heat map showed the correlation between the expression of mitophagy gene mRNA and the activity of the cancer-related Hallmark pathway, red is positive correlation, blue is negative correlation, and the size of the circle represents significance (-log10(P-value))

We further analyzed the protein-protein interaction relationship of MPRGs and drew the protein-protein interaction network (PPI, Protein-Protein interaction). Except for LRBA, we found protein interactions among the remaining eight genes (Fig. 2A). In addition, we also found that there is a co-expression (Co-expression) relationship between MPRGs at the mRNA level (Fig. 2B). For example, HUWE1 is highly positively correlated with PINK1, VPS13D, and LRBA, but not significantly correlated with TAZ expression. TAZ was negatively correlated with positive regulators of mitophagy such as SLC25A4, SLC25A5, VPS13D, suggesting that TAZ plays an opposite role to other genes in KIRC. Mechanistically, TAZ is negatively correlated with various cancer-related HALLMARK pathways, such as Protein_secretion, Estrogen_response_late, MTORC1_signaling, HEME_Metabolism, bile_acid_metabolism and other pathways. The remaining genes were significantly positively correlated with the above pathways (Fig. 2C). In conclusion, TAZ and other MPRGs may play opposite roles in function and mechanism.

### MPRGs risk characteristic construction

**Fig. 3:**
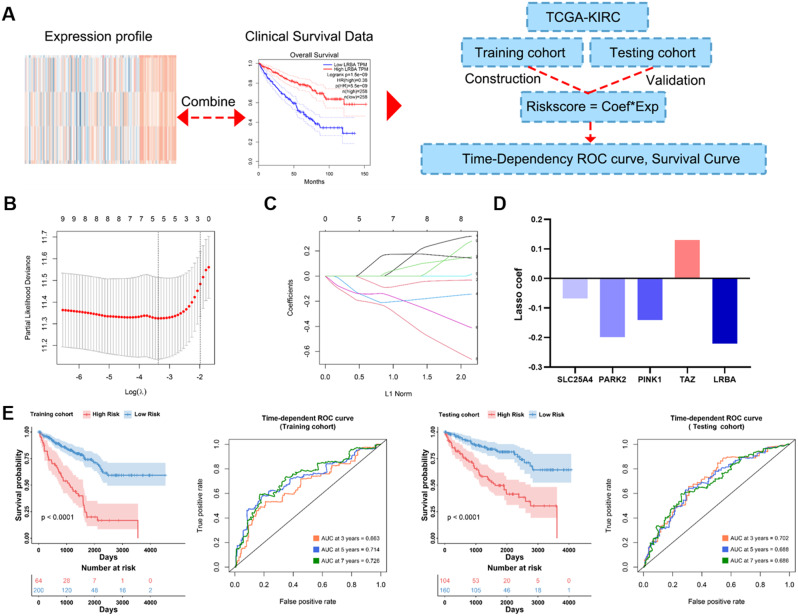
Construction of MPRGs risk characteristics. (**A**) Schematic diagram of the construction and verification of the clinical prediction model; (**B**) Lasso regression analysis process, the picture showed the number of remaining variables when the residual was the smallest; (**C**) Lasso regression analysis process, the picture showed the lasso regression coefficient of different variables; (**D**) Lasso regression coefficients for individual genes of the mitophagy risk signature; (**E**) Survival curve and ROC curve of patients in high and low risk groups in training set and test set

To screen genes closely related to the prognosis of KIRC patients, we combined the mRNA expression profiles of MPRGs with clinical data, and divided KIRC patients into a training set and a test set in a 1:1 ratio. In the training set, Lasso-Cox regression analysis was used to construct the risk feature, and the risk score was calculated based on the expression value of the gene and the lasso regression coefficient, and the test set was used for verification. Further used time-dependent ROC curve and survival analysis to evaluate the accuracy and robustness of risk characteristics (Fig. 3A). The results show that when log(lamda) is equal to 5, the mean square error of the model is the smallest (Fig. 3B). Figure 3C shows the variation of the coefficients of each MPRGs. Ultimately, we identified five MPRGs for constructing the risk score (Fig. 3D), and the formula is as follows:

$$\begin{aligned}{\text{Riskscore}} & \leqslant - 0.06784233*{\text{SLC}}25{\text{A}}4 \\ & \quad - 0.19829176*{\text{PARK}}2 \\ & \quad - 0.14039185*{\text{PINK}}1 \\ & \quad + 0.12976024*{\text{TAZ}} \\ & \quad - 0.22091230*{\text{LRBA}} \\ \end{aligned} $$

Used surv_cutpoint in the survminer package to determine the most appropriate cut-off value (cut-off value), patients above the cut-off value were high-risk group, patients below the cut-off value were low-risk group. We found that in the training and test sets, patients in the high-risk group predicted significantly worse than the lower-risk group (Fig. 3E). The time-dependent ROC curve shows that the risk score has high accuracy in the training set and test set (training set: 3-year AUC = 0.663, 5-year AUC = 0.714, 7-year AUC = 0.726; test set: 3-year AUC = 0.702, 5-year AUC = 0.688, 7-year AUC = 0.686).

### Relationship between MPRGs risk characteristics and clinical variables

**Fig. 4:**
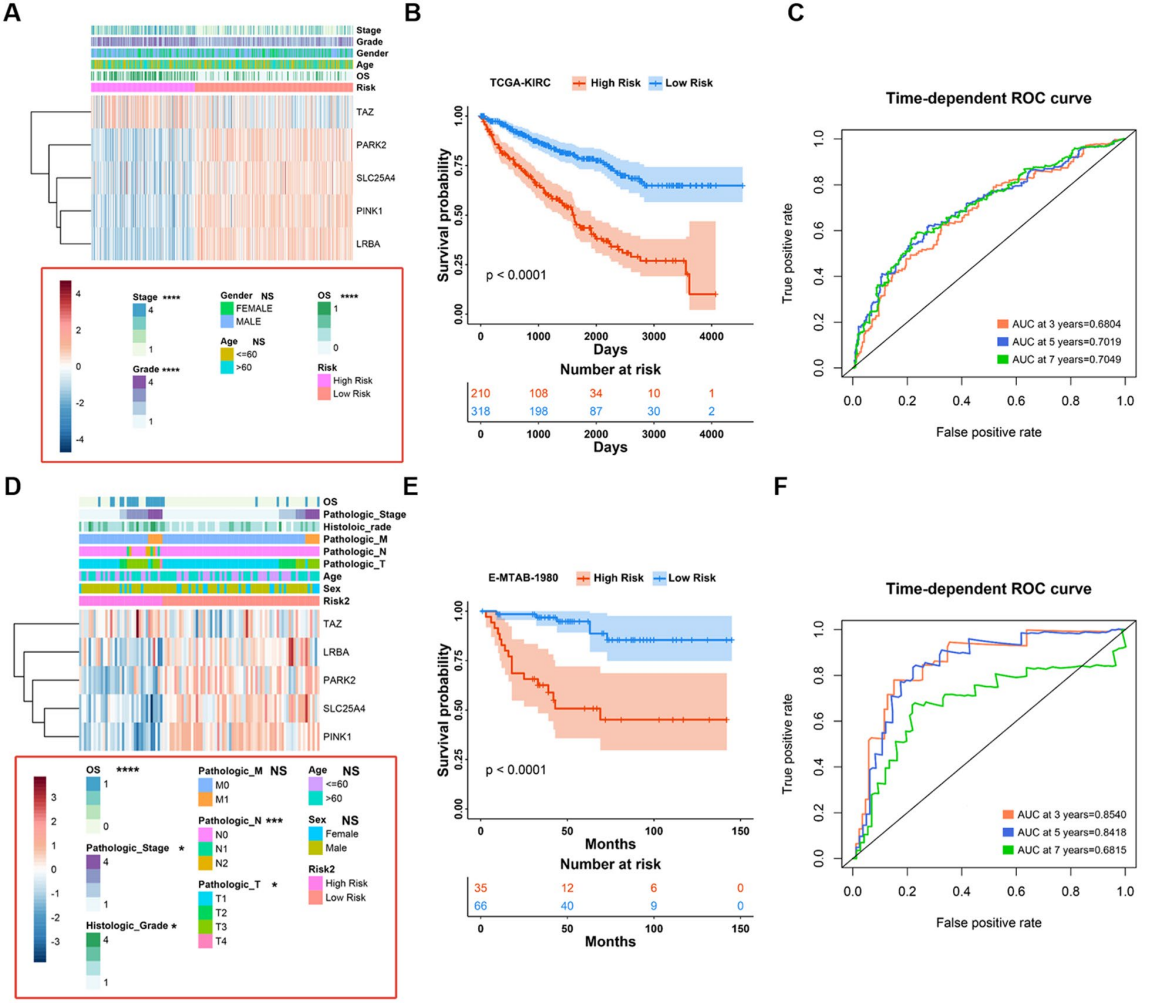
Relationship between MPRGs risk characteristics and clinical variables. (**A**) Distribution of clinical characteristics (prognosis, gender, grade, grade, etc.) and expression of risk signature genes in TCGA-KIRC patients in high- and low-risk groups; (**B**) Kaplain-Meier survival curves of patients in TCGA-KIRC high and low risk groups; (**C**) Accuracy of Mitophagy Risk Score in Predicting Prognosis of TCGA-KIRC Patients by ROC Curve; (**D**) Distribution of patient clinical characteristics (prognosis, gender, grade, grade, etc.) and expression of risk signature genes in the high- and low-risk groups in the E-MTAB-1980 dataset; (**E**) Kaplain-Meier survival curves of patients in high and low risk groups in the E-MTAB-1980 dataset; (**F**) Accuracy of Mitophagy Risk Score in Predicting Prognosis of E-MTAB-1980 Patients by ROC Curve

We combined the data from the training and validation sets to further analyze the relationship between MPRGs risk scores and clinical variables. Across the entire TCGA-KIRC cohort, we found that high-risk patients were associated with higher pathological stage and histological grade, independent of age and sex (Fig. 4A). In addition, the number of dead patients in the high-risk group was more than that in the low-risk group, and the expression of TAZ was significantly high in the high-risk group, and the expression of PARK2, SLC25A4, PINK1, and LRBA were significantly low, which was consistent with the results in Fig. 1F (Fig. 4A). In addition, the prognosis of KIRC patients in the high-risk group was significantly worse, and the risk score showed high accuracy (Fig. 4B and C) (3-year AUC = 0.6804, 5-year AUC = 0.7019, 7-year AUC = 0.7049). The above results were verified in an external validation cohort (E-MTAB-1980 dataset) (Fig. 4D-F). In addition, we divided patients into different clinical subgroups to observe the difference in prognosis of patients in high and low risk groups, and the results were shown in different clinical subgroups (such as cohort of patients older than 60 years and cohort of patients younger than 60 years), patients in the high-risk group all showed worse prognosis. It shows that the risk we constructed has strong robustness (Robustness), and can be used to evaluate the prognosis of KIRC patients in different clinical subgroups (Figure S2A and B).

### Clinical nomogram construction

**Fig. 5:**
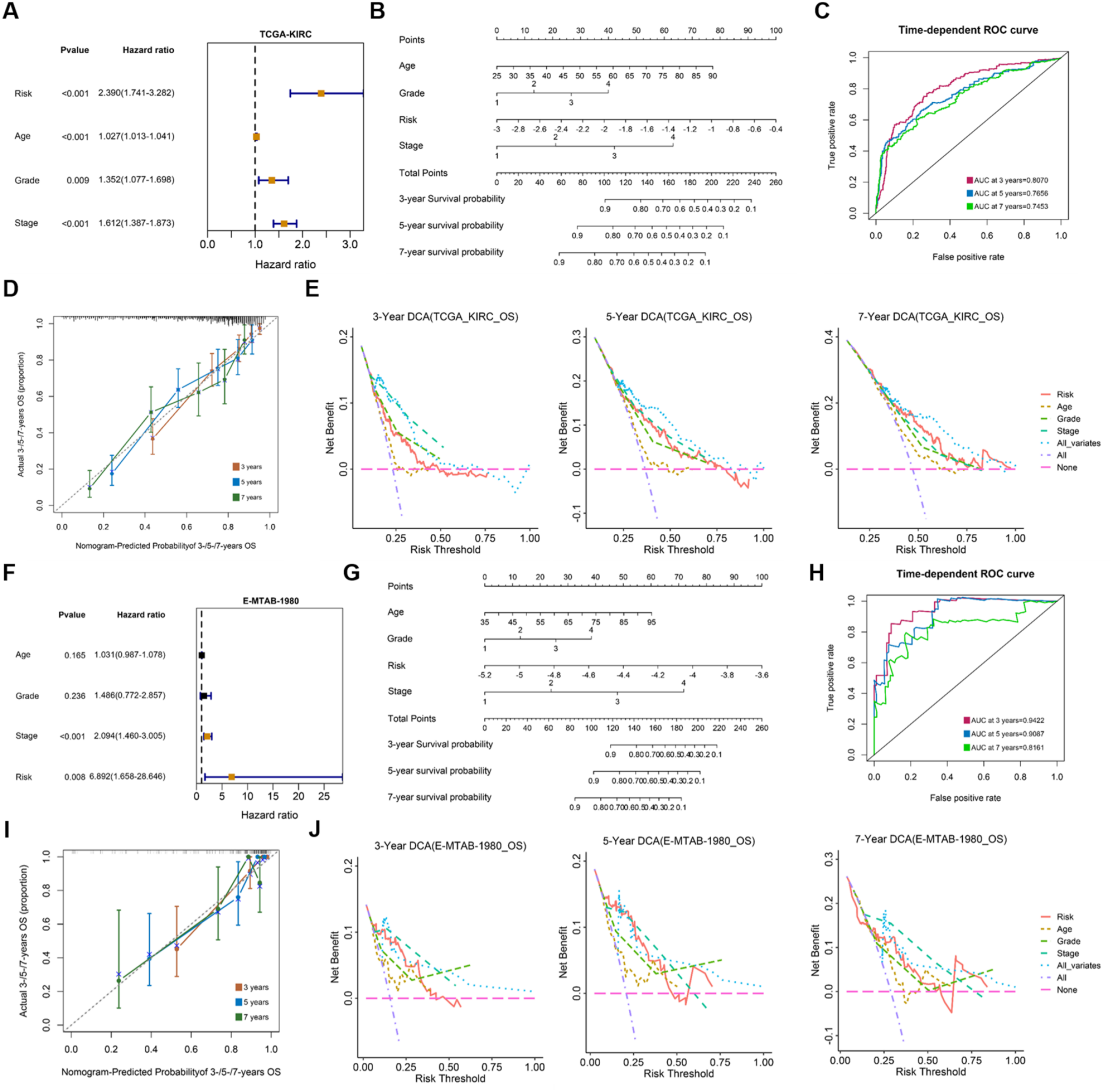
Construct clinical nomogram. (**A** and **F**) Forest plot showed multivariate Cox regression results for age, stage, grade and risk score; (**B** and **G**) Clinical variables (age, stage, grade) and risk score were used to construct a nomogram risk model to predict the patient’s 3-, 5-, and 7-year prognosis; (**C** and **H**) ROC curves showed nomogram model predicting patient 3-, 5-, 7-year prognosis; (**D** and **I**) Calibration curve of nomogram model predicting patient prognosis; (**E** and **J**) Clinical benefit of decision curve analysis comparing single clinical variable and nomogram model in predicting patient prognosis

We further combined the risk score with the four clinical indicators of TCGA-KIRC (Age, Stage, Grade, Gender), and used multivariate Cox regression and stepwise regression to further screen variables. Results showed that risk score, age, stage, and grade were independent prognostic factors, independent of other clinical factors in predicting patient outcomes (Fig. 5A). Therefore, we incorporated these four indicators together into the nomogram model to predict the 3-year, 5-year, and 7-year survival possibilities of patients (Fig. 5B). Time-dependent ROC curve, calibration curve (calibration curve) and decision curve analysis (DCA) showed that these four indicators were more accurate in predicting patient prognosis (3-year AUC = 0.8070, 5-year AUC = 0.7656, 7-year AUC = 0.7453) than any one indicator alone, and the clinical benefit was higher (Fig. 5C-E). In the E-MTAB-1980 cohort, we performed the same analysis and the results were consistent with the TCGA cohort (3-year AUC = 0.9422, 5-year AUC = 0.9087, 7-year AUC = 0.8161) (Fig. 5E-J).

### High expression of TAZ inhibits mitophagy in KIRC

**Fig. 6:**
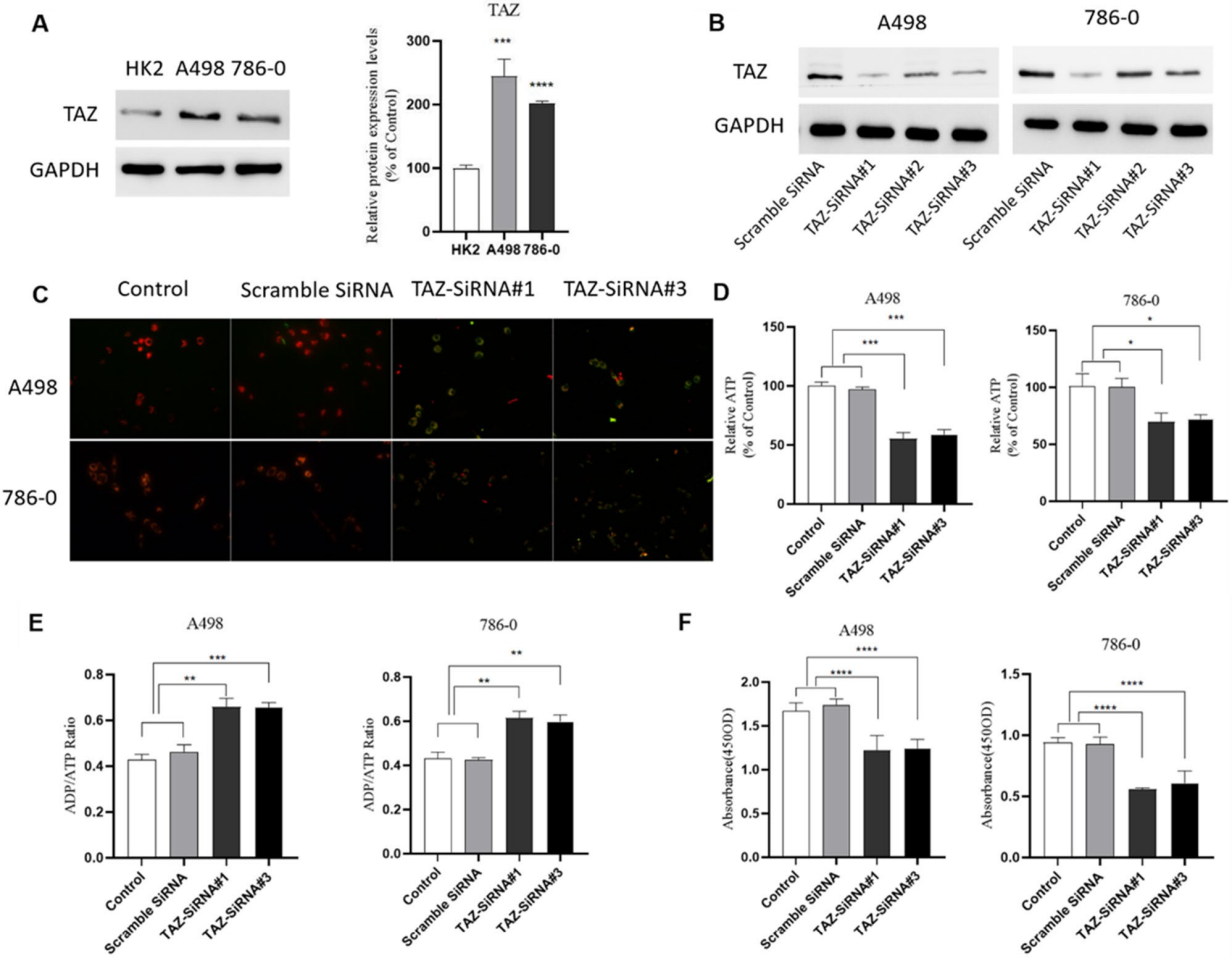
High expression of TAZ inhibits mitophagy in KIRC cells. (**A**) TAZ expression and quantification in different kidney cells (HK2, A498, 786-O); (**B**) Western Blot validate siRNA knockdown of TAZ; (**C**) JC-1 was used to detect the effect of different expression of TAZ on mitochondrial membrane potential; (**D**) Determination of the effect of different expression of TAZ on ATP content; (**E**) Determination of the effect of different expression of TAZ on ADP/ATP ratio; (**F**) CCK8 assay was used to detect the effect of TAZ knockdown on cell viability

To explore the effect of TAZ on mitophagy in KIRC, we selected human renal cortical proximal tubular epithelial cell (HK2) and two types of KIRC cells (A498, 786-O) with different malignant degrees for follow-up research. We found that the TAZ-encoded protein was expressed at a low level in HK2, but at a high expression level in A498 and 786-O (Fig. 6A). To explore whether TAZ plays a role through mitophagy, we first knocked down TAZ in A498 and 786-O (Fig. 6B). The depolarization of damaged mitochondria and the loss of membrane potential are one of the hallmarks of mitophagy. To explore the effect of TAZ expression level on mitophagy, we performed mitochondrial membrane potential detection experiments, and the results showed that compared with the control group, the mitochondrial membrane potential of the TAZ knockout group was significantly reduced (Fig. 6C). We detected the ATP content and ADP/ATP Ratio, and the results showed that the ATP content in the TAZ knockdown group decreased (Fig. 6D), while the ADP/ATP Ratio increased significantly (Fig. 6E). These results indicated that TAZ knockout led to disorder of ATP metabolism, increased mitochondrial damage, and promoted mitophagy. To investigate the effect of TAZ on the viability of KIRC cells, we found that the cell viability in the TAZ knockout group was significantly reduced by CCK8 assay (Fig. 6F). In summary, our results confirmed that high expression of TAZ inhibits mitophagy in KIRC cells, and after knockout of TAZ increased mitochondrial damage and ATP metabolism disorders, thereby promoting mitophagy.

## Discussion

Mitochondria are highly dynamic double-membrane organelles involved in a wide range of biological processes such as ATP production, lipid metabolism, and generation of reactive oxygen species (ROS) [11]. Link between mitochondrial dysfunction and cancer isn’t just about metabolism. Since mitochondria are the “competence factories” of the cell, they are often exposed to high levels of ROS, making them susceptible to mitochondrial DNA mutations and protein misfolding [12]. Mitophagy is the process of autophagy in damaged mitochondria, which is mainly induced by mitochondrial membrane depolarization or changes in mitochondrial DNA [13]. Thus, mitochondrial activity can also affect nuclear or mitochondrial DNA expression and mutations, cell migration, death, and epigenetic changes (e.g., methylation) [14]. Under certain conditions, mitophagy can protect cells from apoptosis and promote tumor cell survival. This suggests a complex interaction between mitochondria, autophagy/mitophagy and tumor initiation. At present, more and more studies have focused on the role of mitophagy in cancer pathogenesis and therapeutic targets. However, there is still a lack of relevant research on the relationship between mitophagy and the pathogenesis of KIRC, and it is worthy of further exploration.

Currently, autophagy, including mitophagy, has been shown to be associated with a variety of kidney diseases. A study showed that mitophagy is closely related to acute kidney injury [15]. Loss of ATG5 or ATG7 in renal epithelium can lead to chronic kidney disease in mice [16]. Mitophagy plays multifaceted roles in carcinogenesis and cancer progression. Autophagy has been considered to play a dual role in tumorigenesis. In different cellular environments, autophagy may play diametrically opposite roles, such as tumor suppression and tumor promotion. Functional mitophagy inhibits the accumulation of damaged mitochondria and prevents carcinogenesis. However, once tumors have progressed, mitophagy can serve as a cytoprotective method to promote tumor progression and resist chemotherapy-induced apoptosis [17]. There are currently few studies on the relationship between KIRC and mitophagy, and the biological role of mitophagy in KIRC remains to be explored. In order to study the connection between mitophagy and the pathogenesis of KIRC, we first used the TCGA database to find the differential gene of KIRC, and intersected with the mitophagy gene. The results showed that a total of 9 mitophagy genes were differentially expressed in KIRC. Our results demonstrate for the first time that TAZ is upregulated in KIRC tissues compared to adjacent normal kidney tissues. Survival analysis of KIRC patients through the GEPIA website found that, as shown in Fig. S1, patients in the TAZ high-expression group had a poor prognosis, while patients in the other eight genes with high expression had a better prognosis. Therefore, we focused on the link between TAZ and mitophagy in KIRC.

TAZ has a similar role to the transcriptional coactivator YES-related protein, both of which are Hippo pathway and its downstream effectors, and play important roles in processes such as development and organ regeneration [18]. It has been demonstrated that YAP/TAZ is overactivated in human cancers and that chronic activation of YAP/TAZ triggers cancer development in mice [19, 20]. Fan et al. found that the activation of mitophagy in myeloma cells was associated with the downregulation of YAP/TAZ expression [21]. This indicates that different expression levels of TAZ are likely to affect mitophagy. To screen genes closely related to the prognosis of KIRC patients, we combined the mRNA expression profiles of MPRGs with clinical data, used Lasso-Cox regression analysis to construct risk features in the training set, and calculated the risk score based on the gene expression values and lasso regression coefficients. Finally, we identified five MPRGs for constructing the risk score, as shown in Fig. 3D. Our experimental results confirmed that TAZ was highly expressed in A498 and 786-O, but low in HK2, which again confirmed our results in the TCGA database. By analyzing the protein interactions of MPRGs and mapping the protein-protein interaction network, we found that TAZ is negatively correlated with positive regulators of mitophagy, suggesting that high expression of TAZ in KIRC may inhibit mitophagy. In order to further explore the effect of different expression levels of TAZ on mitophagy, we knocked out TAZ and detected the indicators related to mitophagy. Our results found that after TAZ knockout, the mitochondrial membrane potential was significantly depolarized, the ATP content decreased, the ADP/ATP ratio increased, and the cell viability decreased. These findings suggest that low expression of TAZ promotes mitophagy in KIRC cells, while high expression of TAZ inhibits mitophagy. This is consistent with our analysis of TAZ protein interactions. Taken together, high expression of TAZ in KIRC inhibits mitophagy and promotes KIRC progression.

To analyze the relationship between MPRGs risk scores and clinical variables, we merged the data from the training and validation sets and found that high-risk patients were associated with higher pathological stage and histological grade, regardless of age and gender. We also found that TAZ was significantly highly expressed in patients in the high-risk group, indicating that high expression of TAZ is closely related to the poor prognosis of KIRC. By analyzing the relationship between the risk characteristics of MPRGs and the clinic, we found that the high expression of TAZ is closely related to the poor prognosis of KIRC. This also proves that TAZ has a promoting role in tumor progression [22]. To better predict the prognosis of KIRC patients, we combined the risk score with four clinical indicators (age, stage, grade, gender), and constructed a clinical nomogram to predict the survival possibility of patients in 3 years, 5 years and 7 years. It has been verified that the prediction model of the clinical prognosis of patients with KIRC has strong robustness and accuracy, and can be used to evaluate the prognosis of different clinical subgroups of KIRC patients. There are currently clinical trials combining autophagy inducers and inhibitors for the treatment of KIRC [7]. Therefore, to assess the prognosis of KIRC patients using our clinical prediction model, KIRC patients are stratified according to the prognostic prediction status. For KIRC patients with poor prognosis, combined treatment with mitophagy inducers or targeted silencing of TAZ can be tried, which will be a brand-new and potential KIRC treatment strategy and provide new ideas for the follow-up research of KIRC.

Combined with our findings, high expression of TAZ and inhibition of mitophagy play an important role in the pathogenesis and progression of KIRC. TAZ upregulation is associated with poor prognosis, tumor progression and mitophagy inhibition in KIRC. The clinical prognosis prediction model we constructed provides a new method for the risk assessment of KIRC and provides an important reference for patients and medical decision-making. In conclusion, TAZ may be an important molecular marker of KIRC and is expected to become a new therapeutic direction targeting KIRC.

### Electronic supplementary material

Below is the link to the electronic supplementary material.

**Supplementary Material 1**: Supplement Figure 1 Kaplan-Meier survival curves of KIRC patients

**Supplementary Material 2**: Supplement Figure 2 Differences in the prognosis of high- and low-risk patients in different clinical subgroups

## Data Availability

The datasets used and analyzed during the current study are available from the corresponding author on reasonable request.
