# Supplementary material for: Transcriptomics analysis revealed that TAZ regulates the proliferation of KIRC cells through mitophagy
Source: BMC Cancer. 2024 Feb 19;24:229. doi: 10.1186/s12885-024-11903-9 (PMC10875871; doi:10.1186/s12885-024-11903-9)
Supplement: Supplementary file 1 — Supplementary Material 1: Supplement Figure 1 Kaplan-Meier survival curves of KIRC patients [file 12885_2024_11903_MOESM1_ESM.docx]

**Supplement Figure 1**


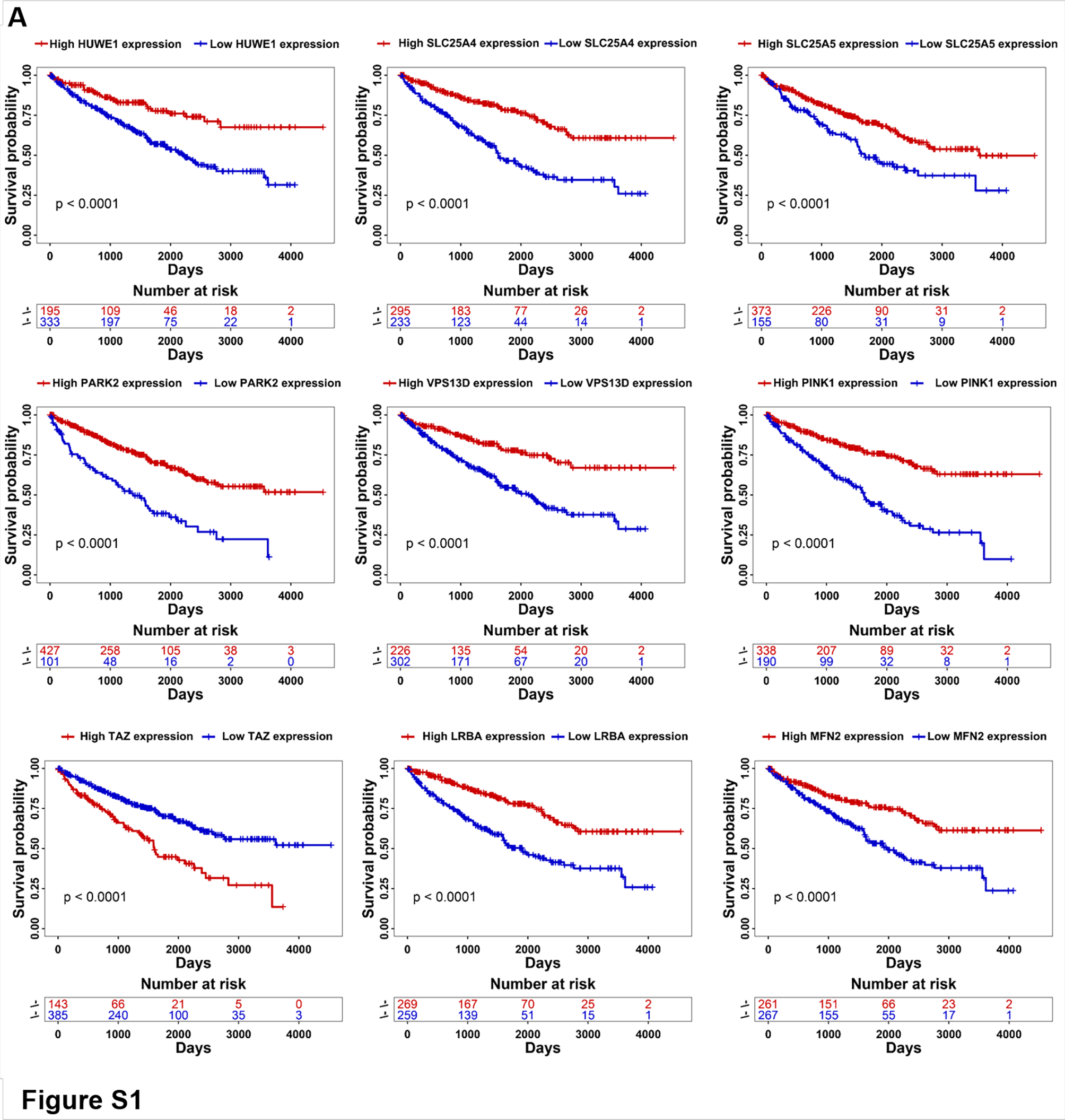


**Supplement Figure 1 Kaplan-Meier survival curves of KIRC patients. (A)** Kaplan-Meier survival curves of mitophagy-related genes.

**Supplement Figure 2**

**
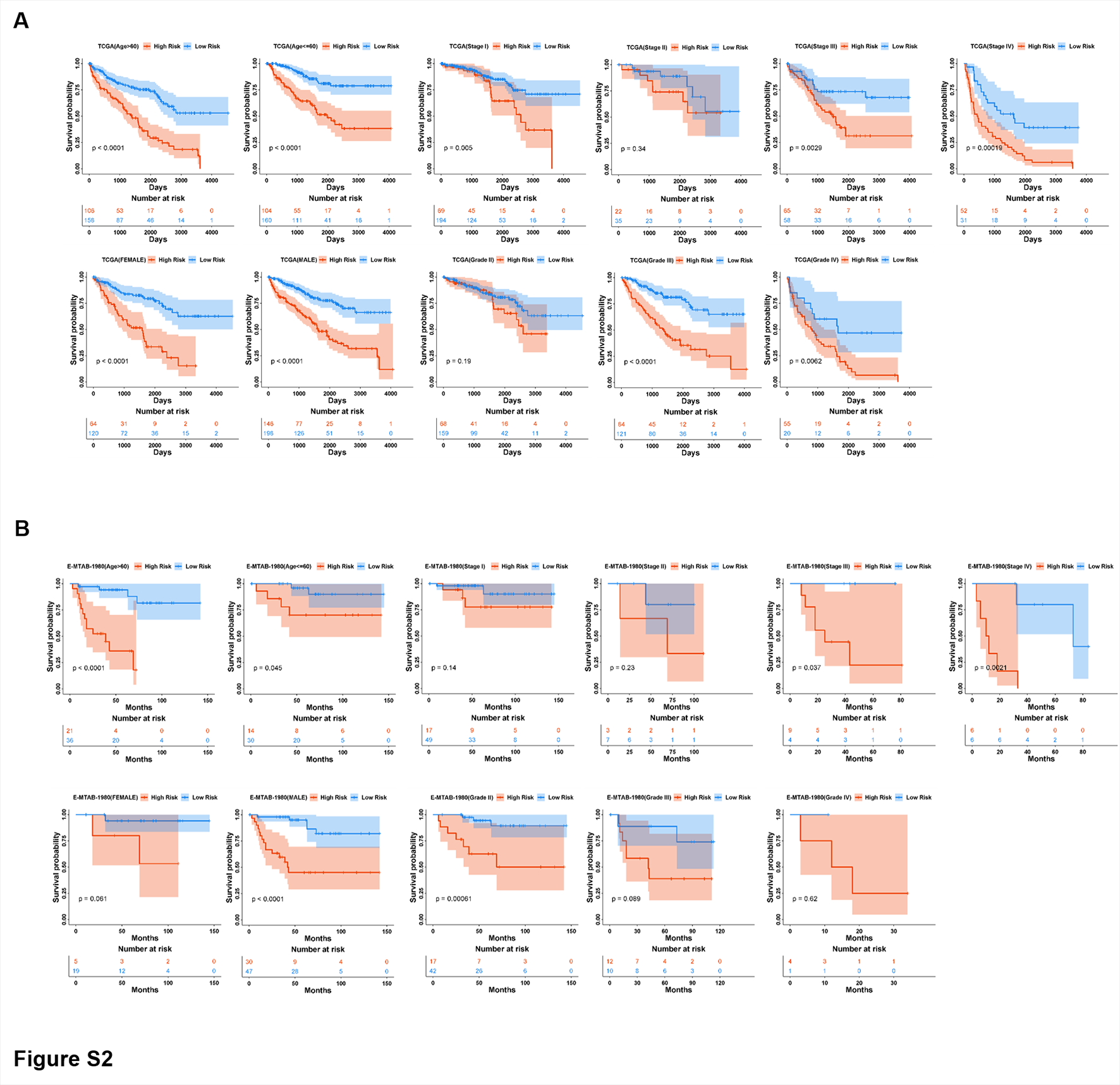
**

**Supplement Figure 2 Differences in the prognosis of high- and low-risk patients in different clinical subgroups. (A and B)** Kaplan-Meier survival analysis revealed differences in prognosis of high- and low-Risk patients in different clinical subgroups (TCGA-KIRC and E-MTAB-1980).
