# Supplementary material for: Transcriptomics analysis revealed that TAZ regulates the proliferation of KIRC cells through mitophagy
Source: BMC Cancer. 2024 Feb 19;24:229. doi: 10.1186/s12885-024-11903-9 (PMC10875871; doi:10.1186/s12885-024-11903-9)
Supplement: Supplementary file 2 — Supplementary Material 2: Supplement Figure 2 Differences in the prognosis of high- and low-risk patients in different clinical subgroups [file 12885_2024_11903_MOESM2_ESM.pptx]

## Slide 1
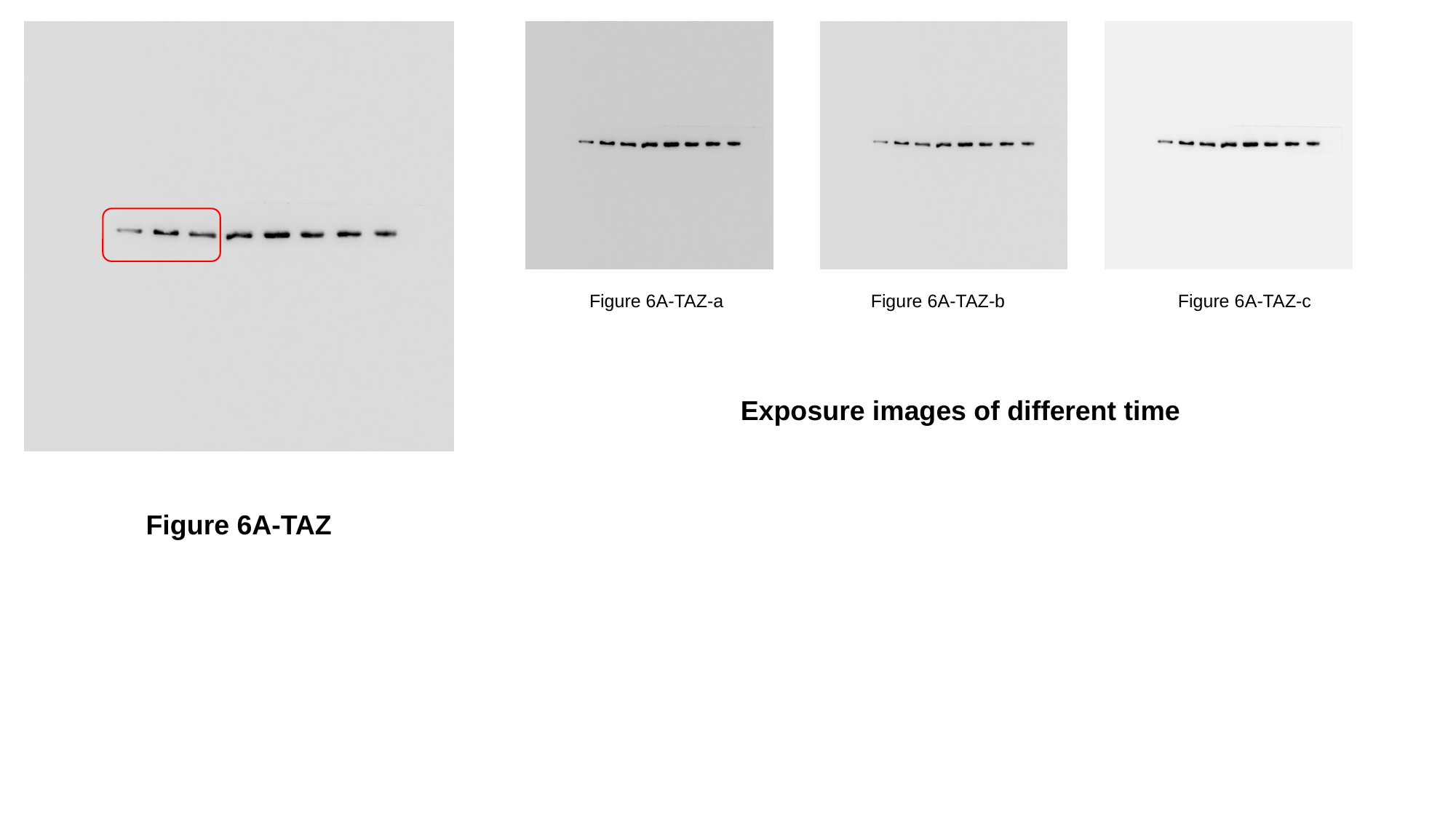

Figure 6A-TAZ-a
Figure 6A-TAZ-b
Figure 6A-TAZ-c
Exposure images of different time
Figure 6A-TAZ

## Slide 2
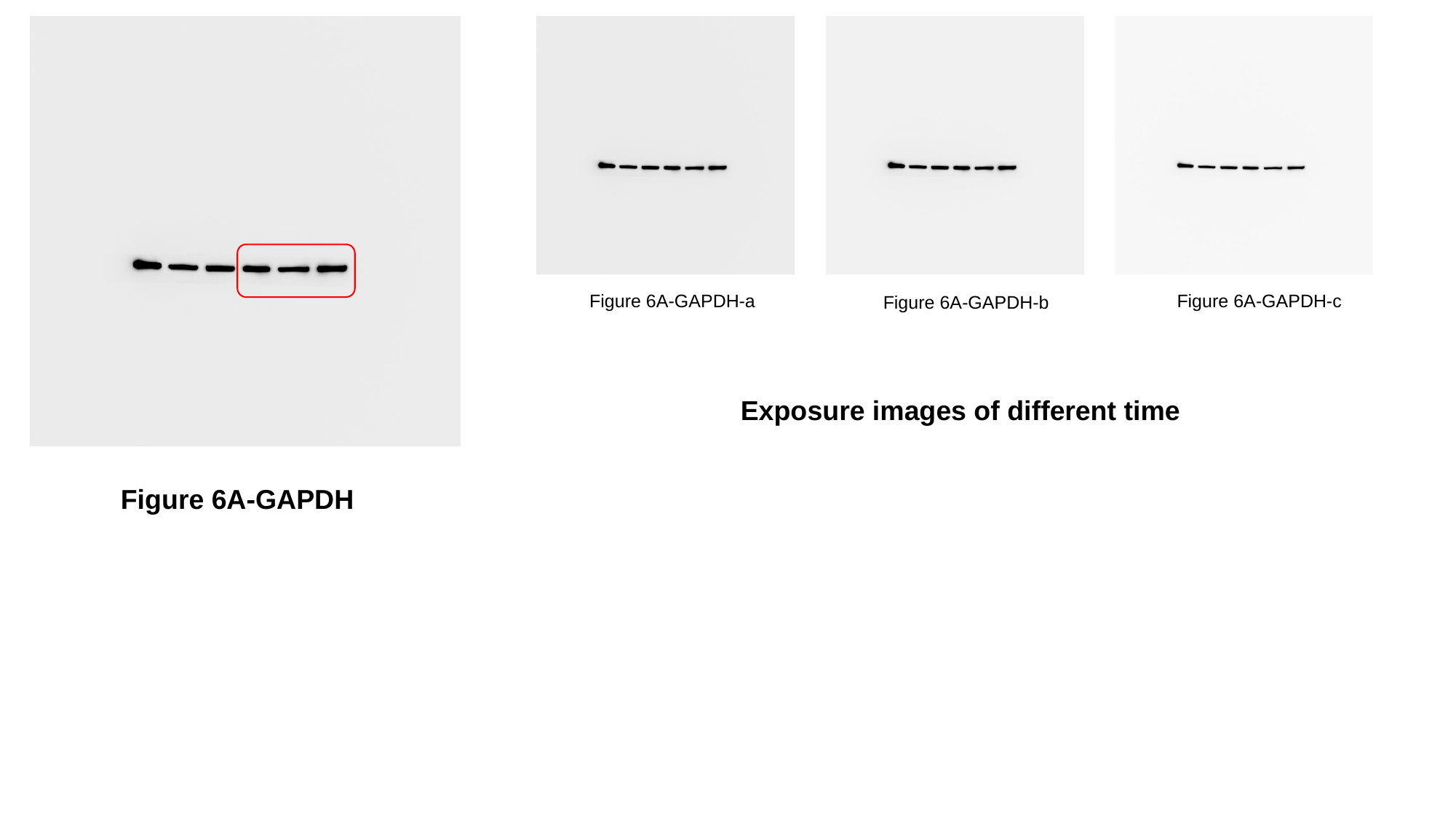

Figure 6A-GAPDH-a
Figure 6A-GAPDH-c
Figure 6A-GAPDH-b
Exposure images of different time
Figure 6A-GAPDH

## Slide 3
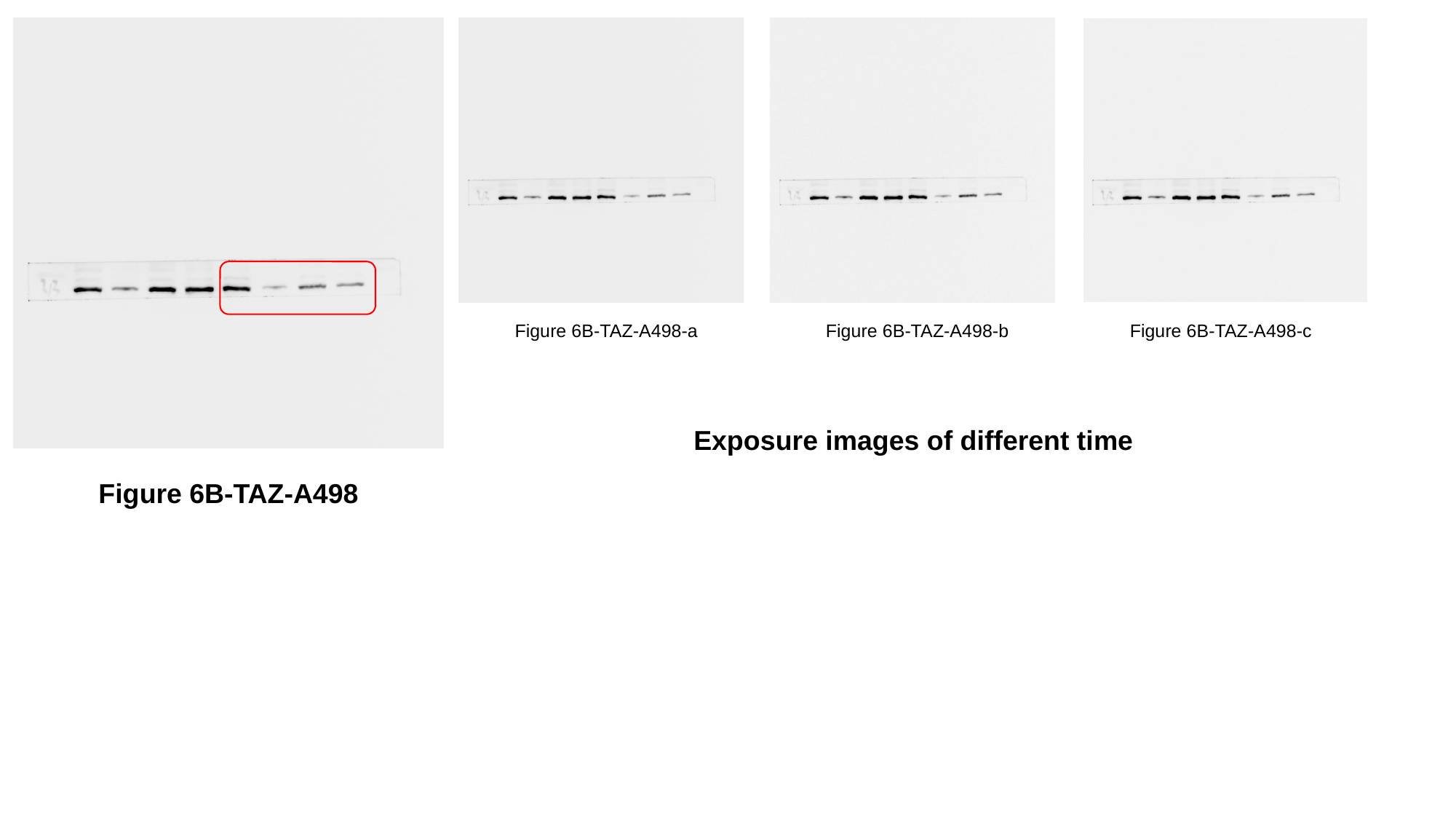

Figure 6B-TAZ-A498-a
Figure 6B-TAZ-A498-b
Figure 6B-TAZ-A498-c
Exposure images of different time
Figure 6B-TAZ-A498

## Slide 4
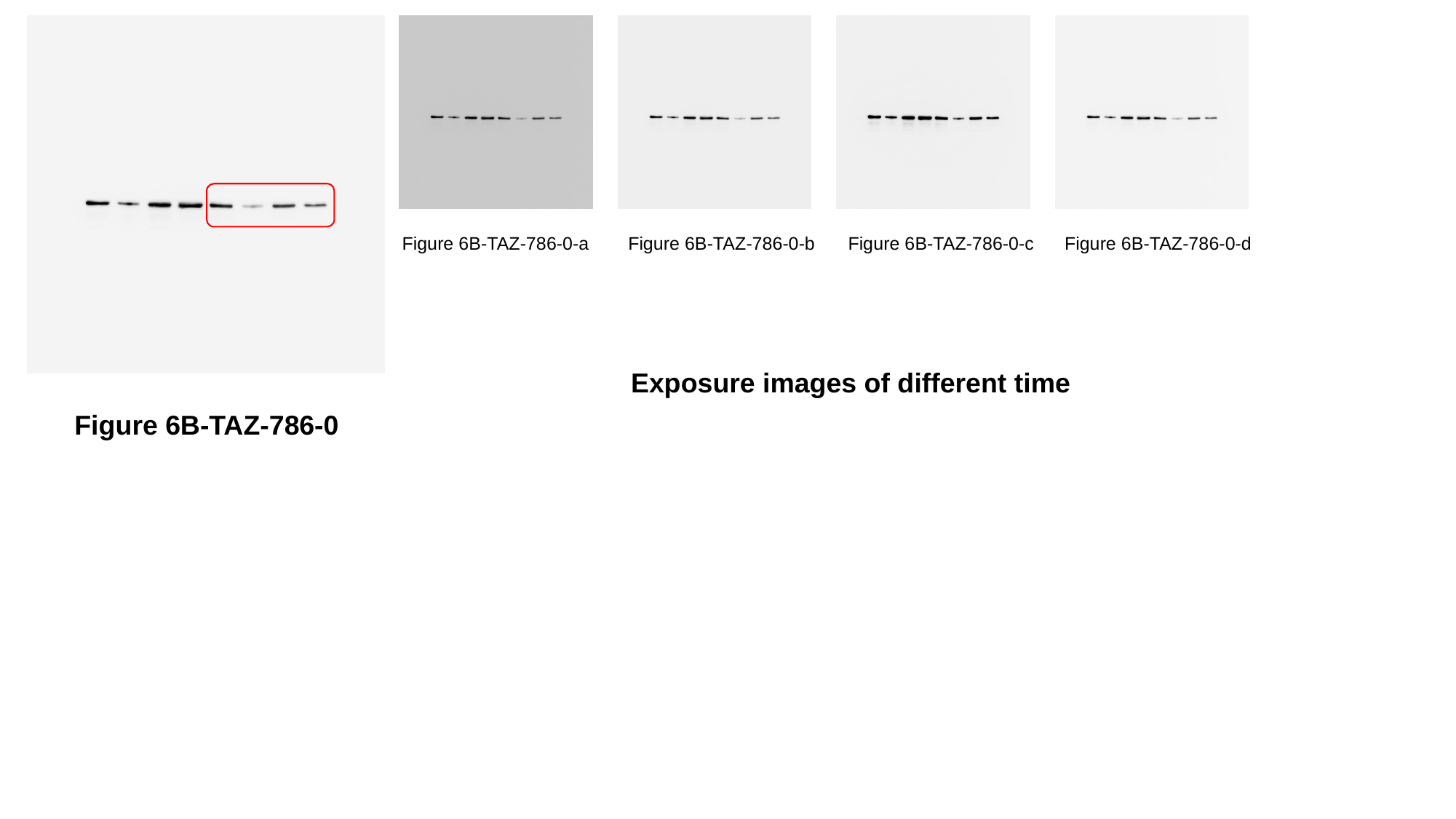

Figure 6B-TAZ-786-0-c
Figure 6B-TAZ-786-0-d
Figure 6B-TAZ-786-0-a
Figure 6B-TAZ-786-0-b
Exposure images of different time
Figure 6B-TAZ-786-0

## Slide 5
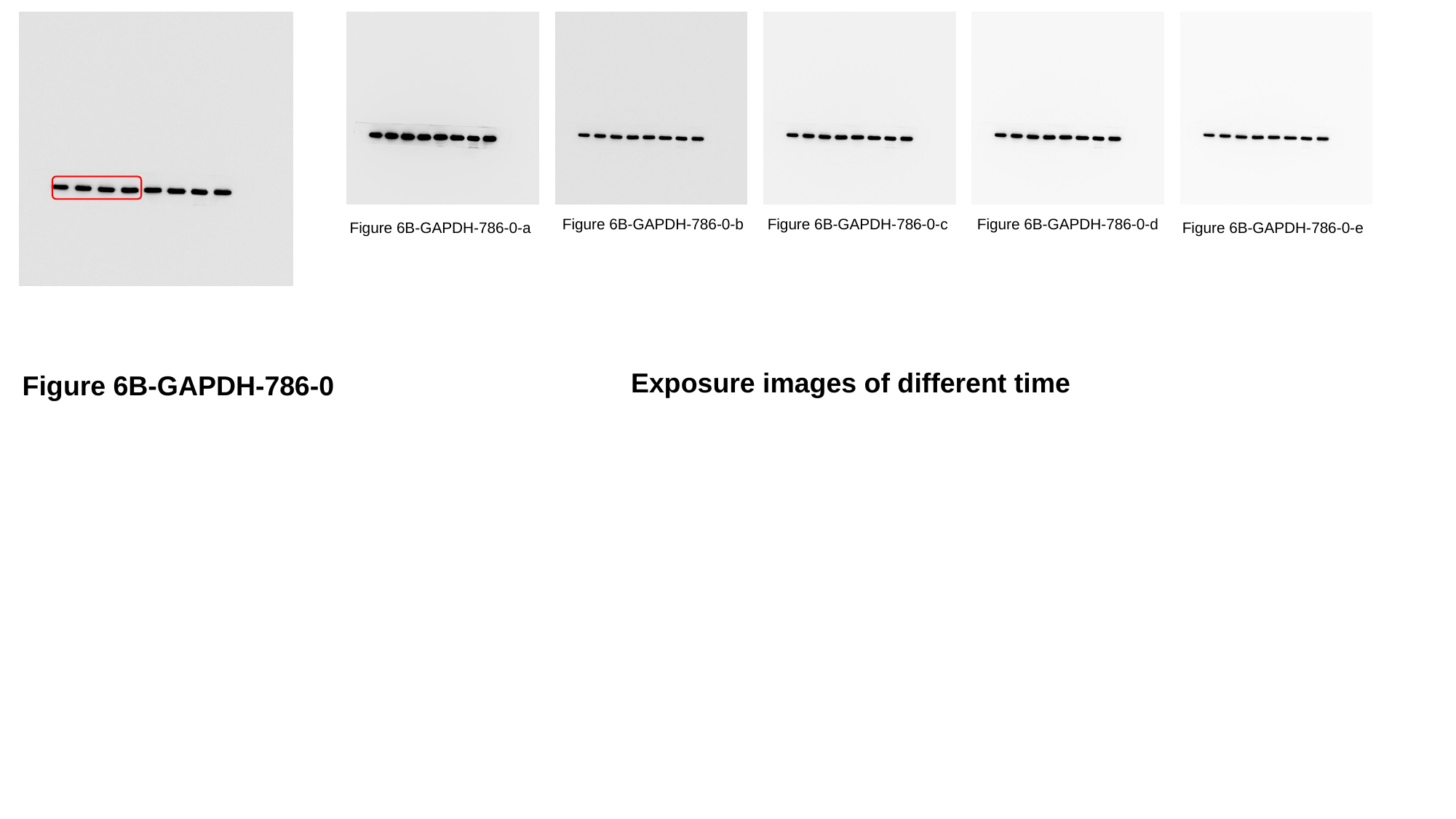

Figure 6B-GAPDH-786-0-d
Figure 6B-GAPDH-786-0-b
Figure 6B-GAPDH-786-0-c
Figure 6B-GAPDH-786-0-e
Figure 6B-GAPDH-786-0-a
Exposure images of different time
Figure 6B-GAPDH-786-0

## Slide 6
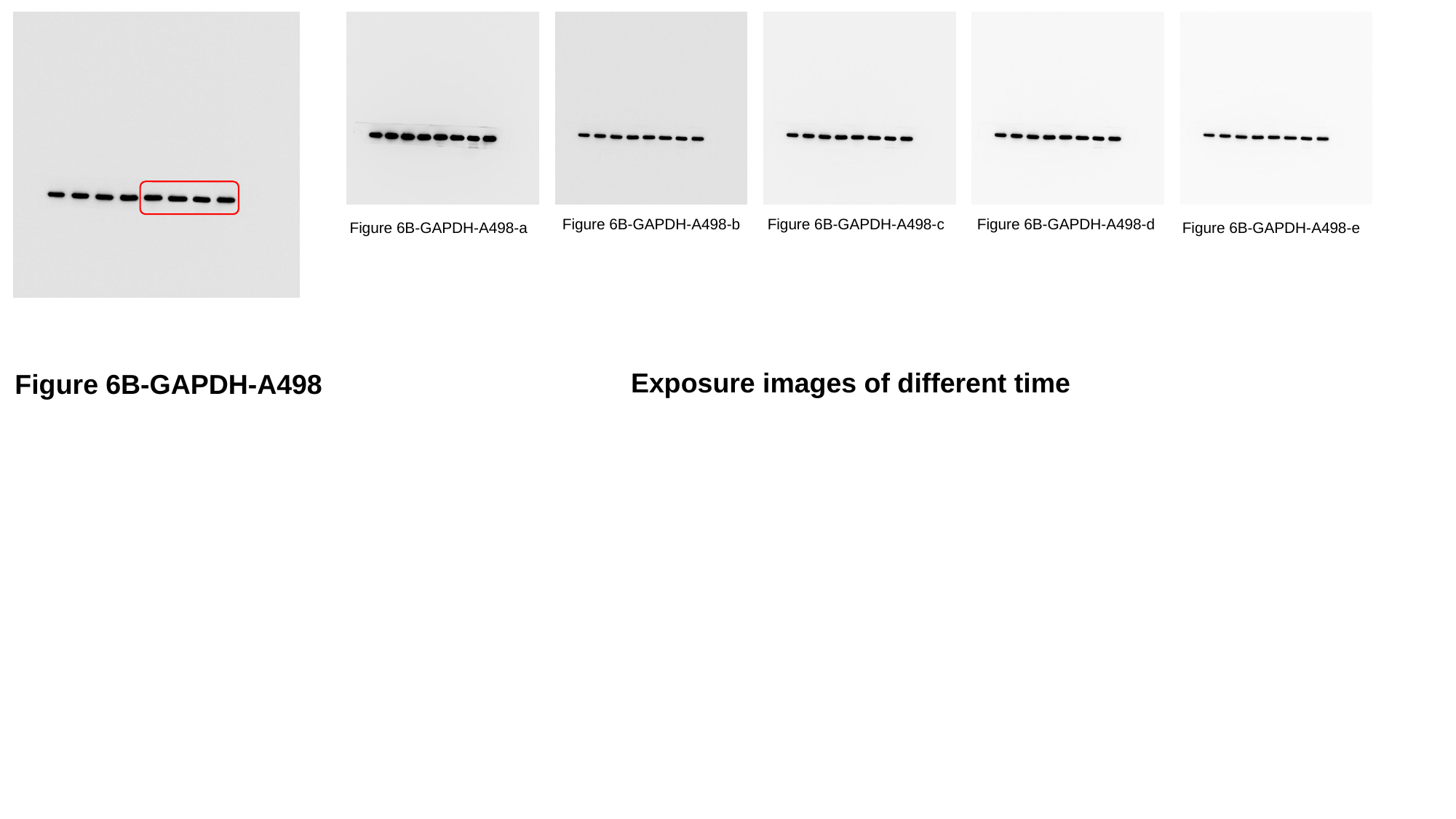

Figure 6B-GAPDH-A498-d
Figure 6B-GAPDH-A498-b
Figure 6B-GAPDH-A498-c
Figure 6B-GAPDH-A498-e
Figure 6B-GAPDH-A498-a
Exposure images of different time
Figure 6B-GAPDH-A498
